# COVID-19 related Delusional Beliefs: A Case Report

**DOI:** 10.1192/j.eurpsy.2023.1715

**Published:** 2023-07-19

**Authors:** S. Akyildirim Cor, B. D. Akcay

**Affiliations:** Psychiatry, Gulhane School of Medicine, Ankara, Türkiye

## Abstract

**Introduction:**

A delusion is a fixed false belief based on an inaccurate interpretation of an external reality despite evidence to the contrary. The diagnosis of a delusional disorder is made when a person has one or more non-bizarre (situations that are not real but also not impossible) delusional thoughts for one month or more that cannot be explained by any other condition. In patients with delusional disorder, delusions(s) do not impact the functionality and the patient’s behavior is not overtly bizarre. Although delusional core themes tend to be the same throughout different epochs (i.e., persecution, grandiosity, guilt, religion, hypochondria, love, or jealous), clinicians commonly notice how delusions tend to rapidly incorporate popular hot topical issues. Hence, delusions are dynamic and often represent a combination of psychopathology and external events.

**Objectives:**

The COVID-19 outbreak has affected millions of people globally and it also has a huge psychological impact. The objective of this case report is to outline the possible effect of the COVID-19 pandemic to delusional disorder in patients with healthy person.

**Methods:**

The 40-year-old gentleman, a drum major (field commander), married, living with his wife and daughter (4,5 years old). He’s current complaints started when he did not want to have the Covid vaccine in April 2021 and therefore was exposed to mobbing at work. It is understood that the patient had irrevocable ideas about vaccine and PCR testing (radioactive lights were coming out from the PCR rod in a video he watched). For this reason, it is understood during the clinical interview that the patient was exposed to social restrictions at work and in his social life (he could not travel by public transport, plane, bus, and enter social facilities because he did not have a vaccination card or did not have a PCR test).He was admitted to our ward for the purpose of arranging his diagnosis and treatment.

**Results:**

On our ward, the patient showed poor insight with persistence of delusions. The impression was delusinal disorder. He was treated with olanzapine up to 5 mg/day and sertraline up to 100 mg/day, with a progressive resolution of symptoms.

**Conclusions:**

There are other case reports on COVID-19 delusional themes in patients with schizophrenia and patients with no history of mental illness, which means that this phenomenon is not exclusive to affective disorders. In this case, different from the literature, the patient has never had covid. An area of clinical concern is the potential of the pandemic’s psychological context to trigger psychotic disorders and influence their symptomatology. A review of contemporary epidemics and pandemics psychosis research found no evidence of changes in the form and content of psychotic symptoms. Further research should examine those biopsychosocial COVID-related factors that predispose to, precipitate, and perpetuate psychosis.

**Disclosure of Interest:**

None Declared

